# The impact of a 6-year comprehensive community trial on the awareness, treatment and control rates of hypertension in Iran: experiences from the Isfahan healthy heart program

**DOI:** 10.1186/1471-2261-10-61

**Published:** 2010-12-21

**Authors:** Alireza Khosravi, Gilda Kiani Mehr, Roya Kelishadi, Shahin Shirani, Mojgan Gharipour, Aliakbar Tavassoli, Fatemeh Noori, Nizal Sarrafzadegan

**Affiliations:** 1 High blood pressure department, Isfahan Institute of Cardiovascular Research, Isfahan University of Medical Sciences, Isfahan, Iran; 2 Preventive Pediatric Cardiology Department, Isfahan Institute of Cardiovascular Research, Isfahan University of Medical Sciences, Isfahan, Iran; 3 Chancellor of Isfahan University of Medical Sciences, Isfahan, Iran; 4 Academic Evaluation and Scientometry Unit, Isfahan Institute of Cardiovascular Research, Isfahan University of Medical Sciences, Isfahan, Iran; 5 Cardiology Department, School of Medicine, Isfahan University of Medical Sciences, Isfahan, Iran; 6 Epidemiology and statististics unit, Isfahan Institute of Cardiovascular Research, Isfahan University of Medical Sciences, Isfahan, Iran; 7 Isfahan Institute of Cardiovascular Research, Isfahan University of Medical Sciences, Isfahan, Iran; 8 Isfahan Institute of Cardiovascular Research, WHO - Collaborating Center for Research and Training in Cardiovascular Diseases Control, Isfahan University of Medical Sciences, Isfahan, Iran

## Abstract

**Objectives:**

We aimed to evaluate the changes over time in the prevalence, awareness, treatment, and control rate of hypertension in intervention and reference areas of a comprehensive community trial with reference area.

**Methods:**

Data from independent sample surveys before and after implementation of the program (2001 vs.2007) were used to compare differences in the intervention and references areas over time. Hypertension was defined as blood pressure ≥140/90 mmHg in non-diabetic patients and ≥130/80 mmHg in diabetic individuals and or taking antihypertensive medications. Interventional activities included educational strategies at population level as well as for hypertensive patients, their families and health professionals.

**Results:**

The study population of the baseline survey included 6175 (48.7% males) in the interventional area and 6339 (51.3% male) in the reference area. The corresponding figures in the post-intervention phase was 4717 (49.3% male) in the interventional area and 4853 (50.7% male) individuals in the reference area. The prevalence of hypertension had a non-significant decrease from 20.5%to 19.6%, in the interventional area whereas in the reference area, it increased from 17.4% to 19.6% (P = 0.003). If we consider Bp ≥ 140/90 in diabetic and non-diabetic patients as hypertension definition, the prevalence of hypertension in the interventional areas had a non-significant decrease from 18.9% in 2001 to 17.8% in 2007, whereas in the reference area, it had a significant rise from 15.7% to 17.9% (P = 0.002) respectively. Awareness, treatment and control rates of hypertension had better improvement in urban and rural part of the interventional area compared to reference area. The awareness, treatment, and control rates of hypertension increased significantly in the age groups of more than 40 years, as well as in all groups of body mass index in interventional areas without significant change in the reference area. Mean systolic blood pressure of study population in the interventional area decreased from 116.13 ±19.37 to 112.92 ± 18.27 mmHg (P < 0.001) without significant change in reference area.

**Conclusions:**

This comprehensive and integrated program of interventions **was **effective in tackling with the prevalence of hypertension, and may improve the awareness, treatment and control rates of this disorder in a developing country setting.

## Background

Hypertension is a major and common risk factor for premature disability and death [1-3]. It is a leading cause of stroke, congestive heart failure, and other cardiovascular diseases in Western [4-6] and Asian [7] populations. Its control is necessary for the prevention of related adverse effects [8]. Some studies have shown poor awareness and control of hypertension in different populations including Iranian populations. This undesirable level of hypertension control may provide a promising area for aggressive intervention [9].

Previous studies in Iranian population showed that despite improvement in the awareness, treatment and control rate of hypertension the situation is not desirable[9,10]. As a public health response to the escalating trend of chronic non-communicable diseases (NCDs) in Iran, Isfahan Healthy Heart Program (IHHP) was conducted as a comprehensive healthy lifestyle community trial for NCDs prevention and health promotion [10,11]. Its interventions were conducted in two provincial counties of Isfahan and Najaf-Abad and were compared with Arak as reference area. One of the objectives of this program was to increase the awareness, treatment and control rates of hypertension [11].

Given the awareness, treatment and control rates of hypertension are low in Iranians [9] it seems that low community awareness and lack of physician training are important. Therefore, IHHP aimed to increase the awareness rate of community and to improve the level of treatment among hypertensive individuals. Increasing the level of health professional's knowledge and treatment skills about hypertension were another goals in this program. This paper presents the results of the 6-years interventions of IHHP on the prevalence of hypertension as well as the changes of hypertension awareness, treatment, and control in the population of the interventional areas in comparison with the reference area. It also reports these differences based on area of residence, age groups and body mass index (BMI).

## Methods

IHHP was conducted as a community-based program for promoting healthy lifestyle behaviors, and ultimately to prevent NCDs in a developing country setting. It integrates interventions and policies that target the major determinants of NCDs including unhealthy nutrition, smoking, physical inactivity and stress [10,11]. Ethics committee of Isfahan Cardiovascular Research Center, a collaborating center of the World Health Organization, and affiliated to Isfahan University of Medical Sciences and other relevant national regulatory organizations approved this study. Written informed consent was obtained from participants after full explanation of the study protocol. In 2000-2001, the baseline survey was conducted in three counties. Two provincial counties of Isfahan and Najaf-Abad with populations of 1,895,856 and 275,084, respectively, were considered as venues of intervention; and Arak, a provincial county located 375 km northwest of Isfahan with a population of 668,531, as the reference area because of socioeconomic, demographic, health profile similarities to the intervention areas. The populations of the three counties were studied for cardiovascular disease (CVD) related risk behaviors and risk factors.

After the baseline survey in 2000, from 2001 interventions were conducted in the intervention communities, and Arak was monitored for comparative purposes; routine national health activities were continuing in both intervention and reference areas. IHHP was developed as an action-oriented, quasi-experimental demonstration program with simultaneous evaluation and different research studies. Its interventions were conducted through 10 projects with different target groups (Additional file 1). Behavioral variables were determined through annual questionnaire-based surveys on independent samples in intervention and reference communities. Then, in 2007 the whole baseline survey was repeated on an independent random sample of both communities. Individuals were selected using multistage random sampling and were invited to the survey centers for interview, clinical examination and blood sampling. A standardized questionnaire was used to obtain information on demographic and socioeconomic aspects.

A trained team of physicians performed physical examinations using standardized and zero-calibrated instruments for measuring blood pressure(BP) [12]. BP was measured, manually by a trained operator using a mercury sphygmomanometer according to a standard protocol, twice from each right and left arms in sitting position after 5 minutes of rest. The first Korotkoff sound was recorded as the systolic blood pressure (SBP) and the disappearance of the sounds (V phase) was considered as the diastolic blood Pressure (DBP). The values of hypertension used in analysis were recorded as mean measured BP in higher arm [13].

The aims of our interventions are to improve the knowledge, attitude and behaviors of general population, hypertensive patients, their relatives and health professionals and to increase the chance of early detection, treatment and control of hypertension by means of education of health professionals (physicians, nurses and health staff), patients and public about prevention, early diagnosis, adherence to treatment and control of hypertension.

Hypertension was defined as an average BP equal or more than 140/90 mm Hg in non-diabetic patients and equal or more than 130/80 mmHg in diabetic subjects[12] or if the patient was taking antihypertensive medications. Participants, who had been previously diagnosed to have hypertension, were considered to be aware of their hypertension. Participants were considered to be treated if they were taking antihypertensive drugs. Hypertension was considered controlled if the patients were receiving anti-hypertensive medications and their average blood pressure was considered less than 140/90 mm Hg in non-diabetic patients or less than 130/80 mmHg in diabetic patients. Participants were considered to have diabetes whether it had been diagnosed previously by a doctor or they had two separate fasting blood glucose more than 125 mg/dl or 2 hour post-prandial glucose more than 200 mg/dl or if they were receiving anti-diabetic medications [13].

For quality control purposes, the whole IHHP have been extremely evaluated with promising results [11].

### Statistical analysis

Statistical analysis was performed using the SPSS statistical package version 15.0 for Windows (SPSS Inc., Chicago, USA). The significance level was set at P < 0.05. The data are presented as frequencies, percentages and 95% confidence intervals. Prevalence of hypertension, awareness, treatment, and control rates in interventional and reference areas were compared by x² test. The prevalence of hypertension as well as the awareness, treatment, and control rates were adjusted for age, sex and BMI were compared with logistic regression test to calculate the independent association between the above variables and the prevalence, awareness, treatment and control rates of hypertension.

## Results

In this study, data on 10884 participants in interventional counties (Isfahan and Najaf Abad) and 11192 in reference city (Arak) were included in 2001 and 2007 as a whole. The study population of the pre-intervention survey included 6175 in the interventional area and 6339 in the reference area. The corresponding figure in the post-intervention survey was 4717 (47.19% male) in the interventional area and 4853 (50.7% male) individuals in the reference area.

Table 1 shows the age, sex, BMI, BP and diabetes status of the population studied in 2001 and 2007. There was no overall significant difference in mean age in interventional and reference Table 2 demonstrates the prevalence, awareness, treatment and control of hypertension in interventional vs. reference areas in 2001 and 2007. The prevalence of hypertension in the interventional areas had a non-significant decrease from 20.5% in 2001 to 19.6% in 2007, whereas in the reference area, it had a significant rise from 17.4% to 19.6% (P = 0.003) respectively. If we consider blood pressure≥ 140/90 in diabetic and non diabetic individuals for definition of hypertension the prevalence of hypertension in the interventional had no significant decrease from 18.9% in 2001 to 17.8 in 2007, whereas in the reference area, it had a significant rise from 15.7% to 17.9%(p = 0.002). Mean systolic blood pressure in study population decreased significantly in interventional area (P < 0.001) whereas in the reference population, this decline was not significant (P = 0.24). Mean diastolic blood pressure decreased in both interventional and reference areas but this reduction was significant in reference area.

**Table 1:** Characteristics of participants: IHHP program

		Interventional area			reference area		
		
		Before (N = 6175)	After (N = 4719)	P-value	Before (N = 6339)	After (N = 4853)	P-value
**Age year (mean±SD)**		38.61 ± 14.72	38.54 ± 15.27	0.83	39.17 ± 15.12	39.03 ± 15.85	0.64
**Sex**	**Male**	48.7%	49.3%		49.2%	50.7%	
	**Female**	51.3%	50.7%	0.51	50.8%	49.3%	0.12
**BMI (kg/m^1)^**	**<25**	45.7%	44.5%		53.1%	49.4%	
	**25-<29.9**	35.4%	37.0%	0.23	32.6%	34.9%	0.001
	**30**	18.9%	18.5%		14.3%	15.6%	
**Diabetes mellitus**		6.3%	7.1%	0.09	4.9%	6.5%	<0.001
**Prevalence of hypertension** **base on DM(2)**		20.5%	19.6%	0.24	17.4%	19.6%	0.003
**Prevalence of hypertension(3)**		18.9%	17.8%	0.14	15.7%	17.9%	0.002
**Mean systolic blood pressure**		116.13 ± 19.37	112.92 ± 18.27	<0.001	115.22 ± 18.96	114.79 ± 19.62	0.24
**Mean diastolic blood pressure**		74.79 ± 11.93	74.43 ± 11.06	0.11	76.18 ± 10.22	73.54 ± 11.03	<0.001

1) Body mass index (kg/m2)

2) BP ≥ 140/90 in nondiabetic BP ≥ 130/90 in diabetic patient

3) BP ≥ 140/90 in diabetic & nondiabetic patient

**Table 2:** Awareness, treatment and control rates of hypertension in interventional and reference area: IHHP, 2001-2007

Time Area		Awareness			Treatment			Control		
	
		2001 N = 12514	2007 N = 9572	P-value	2001 N = 12514	2007 N = 9572	P-value	2001 N = 12514	2007 N = 9572	P-value
Interventional area	Urban	404(41.6)	349(48)	0.009	341(35.2)	305(42.5)	0.002	77(7.9)	112(15.6)	<0.001
	
	Rural	107(36.5)	92(58.2)	<0.001	100(34.2)	78(49.7)	0.001	12(4.1)	26(16.6)	<0.001
	
	Total	511(40.4)	441(49.8)	<0.001	441(34.9)	383(43.8)	<0.001	89(7.1)	138(15.8)	<0.001

Reference Area	Urban	314(44.1)	212(48.5)	0.15	267(37.5)	190(43.8)	0.035	78(11)	71(16.4)	0.009
	
	Rural	143(37.1)	232(45.2)	0.015	121(31.5)	188(36.9)	0.096	29(7.6)	61(12)	0.031
	
	Total	457(41.7)	444(46.7)	0.021	388(35.4)	378(40)	0.031	107(9.8)	132(14)	0.003

The awareness, treatment and control of hypertension increased significantly in rural and urban areas of interventional countries (P < 0.001), the awareness and treatment were not significant in the reference area. (Table 2)

Table 3 shows that in interventional areas, the awareness, treatment, and control rates of hypertension increased significantly in the age groups of more than 40 years (*P <*0.01). In the age group of 19-39 years old no significant increase was seen in the awareness, treatment, and control rates of hypertension. This table also shows that the awareness, treatment and control rates of hypertension had a more favorable improvement in interventional than in reference area. These rates had significant improvement in population aged ≥40 years in interventional vs. reference areas too. Awareness, treatment and control rates of hypertension increased significantly in all groups of BMI in interventional area the corresponding figures were not significant in the reference area.

**Table 3:** Awareness, treatment, and control of hypertension by age and body mass index in interventional and reference areas: IHHP, 2001-2007

	Percentage (%)					
**Age and BMI**	**Interventional area**			**Reference area**		
	****2001**** **N = 12514**	****2007**** **N = 9572**	****P-value****	****2001**** **N = 12514**	****2007**** **N = 9572**	****P-value****

Awareness						
Age, y						
19-39	47(18.0)	43(24.4)	0.10	26(13.1)	39(20.9)	0.043
40-59	206(37.9)	183(48.8)	0.001	183(39.8)	140(41.7)	0.59
≥60	258(56.2)	215(64.4)	0.021	248(56.5)	265(62.1)	0.095
BMI, kg/m2						
<25	93(29.2)	76(41.1)	0.007	146(38.3)	138(43.0)	0.209
25-29.9	216(40.8)	192(49.2)	0.011	180(41.8)	191(47.9)	0.077
≥30	192(48.6)	163(56.2)	0.049	130(45.9)	111(49.8)	0.39
Treatment						
Age, y						
19-39	33(12.7)	29(16.6)	0.26	19(9.6)	34(18.2)	0.015
40-59	173(31.8)	154(42.0)	0.002	151(32.9)	115(34.3)	0.67
≥60	235(51.3)	200(60.2)	0.013	218(49.7)	229(54.3)	0.18
BMI, kg/m2						
<25	87(27.4)	70(37.8)	0.014	125(32.9)	118(36.9)	0.27
25-29.9	188(35.6)	161(42.1)	0.045	148(34.3)	160(40.4)	0.071
≥30	156(39.5)	142(49.5)	0.009	114(40.3)	96(43.4)	0.48
Control (all treated)						
Age, y						
19-39	13(5.0)	16(9.1)	0.089	11(5.6)	21(11.2)	0.044
40-59	30(5.5)	56(15.3)	<0.001	42(9.2)	44(13.1)	0.078
≥60	46(10.0)	66(19.9)	<0.001	54(12.3)	67(15.9)	0.13
BMI, kg/m2						
<25	20(6.3)	24(13.0)	0.011	41(10.8)	47(14.7)	0.12
25-29.9	43(8.1)	63(16.5)	<0.001	42(9.8)	54(13.6)	0.083
≥30	24(6.1)	46(16.0)	<0.001	24(8.5)	31(14.0)	0.048

Multiple logistic regression analysis revealed that after adjustment for the residence area and the year of study, the prevalence of hypertension in the age groups of 40 to 59 years and more than 60 years were 4.64 and 18.30 times more than the age group of 19 to 39 years. In addition, the prevalence of hypertension was 1.98 and 3.14 times higher in overweight and obese individuals in comparison with normal-weight participants. In the age groups of 40 to 59 years and more than 60 years the awareness about hypertension was 2.89 and 6.99 times higher than in the age group of 19 to 39 years. Furthermore, overweight and obese individuals were 1.35 and 1.68 times more awarded of their high blood pressure than normal weight population. Multiple logistic regression analysis revealed that the treatment of hypertension in age groups of 40 to 59 years and more than 60 years were 3.15 and 7.87 times higher than in the age group of 19 to 39 years. In addition, treatment rates were 1.22 and 1.50 times higher in overweight and obese individuals than in normal weight participants. Hypertension control was 1.46 and 2.26 times higher in the age groups of 40 to 59 years and more than 60 years than the age group of 19 to 39 years. Also it showed that the prevalence, awareness, treatment, and control rate of hypertension in high school diploma patients were better than lower educated patients. Overweight individuals had the same control rate as normal weight individuals but obese individuals had low control rate (0.86%) of hypertension in comparison with normal weight individuals. The awareness, treatment and control rates of hypertension were 2.22, 2.3, and 2.37 times more in women compared to men and in educated population it is 1.11, 1.18, and 1.61 times higher than non-educated participant (Table 4).

**Table 4:** Multiple logistic regressions of prevalence, awareness, treatment and control of hypertension in the interventional and reference areas: IHHP, 2001-2007

Predictors	prevalence	Awareness	Treatment	Control
**Age **(reference: 19-39 y)	**R**	**R**	**R**	**R**

**40-59 y**	4.64(4.22-5.1)	2.89(2.35-3.56)	3.15(2.51-3.97)	1.46(1.06-2.01)

**≥60 y**	18.30(16.37-20.46)	6.99(5.62 - 8.69)	7.87 (6.21-9.98)	2.26(1.64-3.11)

**Sex **(reference: man) **Woman**	0.995(0.92-1.08)	2.22 (1.93-2.55)	2.298(1.99-2.65)	2.37(1.89-2.96)

**High school diploma**	0.69(0.63 - 0.77)	1.11(0.91-1.37)	1.18(0.95-1.46)	1.61(1.19-2.18)

**BMI **(reference : <25 kg/m^2^)	R	R	R	R

**Overweight**, 25-29.9 kg/m^2^	1.98(1.80-2.17)	1.35(1.15-1.59)	1.22(1.03-1.45)	1.01(0.798-1.29)

**Obese**, ≥30 kg/m^2^	3.14(2.82-3.50)	1.68(1.397-2.01)	1.497(1.24-1.80)	0.86(0.66-1.13)

Adjusted for years and residency (intervention area and reference area)

## Discussion

In this community trial, that to the best of our knowledge is the first of its kind in developing countries, the integrated and comprehensive interventions of this population-based program resulted in favorable changes in the prevalence, awareness, treatment and control of hypertension. Although the decline in the prevalence of hypertension was not significant in the intervention countries, but by considering its significant increase in the reference country while usual health care services were similarly undergoing in both areas. It can be assumed that if specific interventional programs were not performed in interventional areas, the prevalence of hypertension would have increased similar to the reference area. Moreover, the mean systolic BP declined significantly in the intervention population but not in the reference area.

Previous studies in the US have shown that the prevalence of hypertension to be 28.7%, with an increase of 3.7% from 1988 to 1991 Hypertension. The prevalence of hypertension was highest in non-Hispanic blacks (33.5%), increased with age (65.4% among those aged ≥60 years), and tended to be higher in women (30.1%) [14]. In another study that was conducted in the US in 2003-2004 the overall prevalence was 29.3%. When compared with 1999-2000, there was no significant increase in the overall prevalence of hypertension [15]. A study in China revealed that the overall prevalence of hypertension was 48.5% [7].

Reduction in BP can lead to a decline in the high morbidity and mortality rate in individuals with hypertension [15]. Therefore, in the current study, 3.18 mmHg reductions in systolic BP in interventional areas may result to a significant decline in the incidence of heart failure, stroke, myocardial infarction and other atherosclerotic events in the future [15]. The reason behind this decline in interventional areas might be increasing the awareness of population about hypertension and simultaneous education of health professionals on the role of pharmacological and non pharmacological treatment as well as how to treat patient with multiple anti-hypertensive drugs and efforts to increase the patients' compliance. The synergetic and dose of interventional activities in such comprehensive program might be effective as well.

While no change was observed in interventional areas, a significant decrease was seen in mean diastolic pressure in reference area. Changes in diastolic blood pressure in the population's studies are difficult to explain, whereas we educated separate personnel in interventional and reference area to measure diastolic BP. it may be due to more difficult and less accurate measurement of the diastolic blood pressure based on Korotkoff sounds in the general population. Other studies showed controversial changes in diastolic pressure during different periods of the study [15,16].

Some studies showed Prevalence of diabetes in adults worldwide was estimated to be 4.0% in 1995 and will rise to 5.4% by the year 2025[15]. It is higher in developed countries than in developing ones. This increase is probably due to population aging, urbanization, and increase in the prevalence of obesity and physical inactivity [16]. In our study the prevalence of diabetes mellitus showed a non significant uprising trend in interventional area (from 6.3% to 7.1%) and significant increase in reference area (from 4.9% to 6.5%). This increase in control group was 2 times greater than the interventional group (table 1). Therefore, IHHP activities may have slowed down the rapid uprising trend of diabetes in interventional areas.

Using BP ≥140/90 mm Hg as a cut-off level in hypertensive people with diabetes might underestimate the prevalence of hypertension. In our study the prevalence of diabetes in general population was up to 6.3%, (table 1) so we defined hypertension in diabetic patients equal or more than 130/80 to attain a more accurate prevalence rate [12]. Additionally, studies using a BP goal of <140/90 mm Hg in hypertensive people with diabetes might overestimate BP control rates [17]. Therefore, in this study consistent with a previous study [17], different control goals (<130/80 mm Hg) were used for those with diabetes to provide a more accurate control rate.

Two surveys in England and Canada documented that many individuals with hypertension are unaware of their disease (63% and 42% respectively), and those who are awared are often untreated (19%) or under-treated (23%)[18,19]. In this study the improvement in awareness, treatment and control rates of hypertension in rural and urban areas of interventional areas were the same, both better than reference area. (9.7 vs. 5.1, 8.7 vs. 4.8 and 8.7 vs. 4.4 respectively)(Table 2). This improvement may lead to decrease future cardiovascular events in our country.

Evaluation of the increase in awareness, treatment and control of hypertension in different age groups was another important way for assessing the efficacy and conduction of interventional programs and may need more aggressive intervention in low compliance age groups. The current study demonstrates that since 2001, awareness, treatment and control rates of hypertension in patients who are older than 40 years in interventional areas increased significantly (table 3) and this might be due to their greater concern about their health status or less effectiveness of interventional activities in younger people. In spite of non-significant rise in awareness, treatment and control of hypertension in younger patients we had a favorable trend in this group; therefore more specific programs may be needed for this age group in the future. Furthermore, previous studies such as NHANES study showed that, compared with younger individuals with hypertension, older individuals with hypertension have a lower control rate despite being equally likely to be treated[20-22]. Other study reported that the awareness and control rates of hypertension were better in older people, whereas there was a relatively under-treatment for the case of hypertensive people of younger age [14]. In our study awareness, treatment and control rates of hypertension improved significantly in normal weight, over weight and obese patients; in interventional area the corresponding figures were not significant in the reference area.

In our study, in all participants (interventional and reference areas) the number of hypertensive patients with optimal weight (BMI< 25 kg/m^2^) decreased but in the reference area the prevalence of over-weight and obesity increased from 2001 to 2007 compared to interventional area. This might be the result of the effects of interventional activities in interventional areas. This difference in shifting BMI in interventional and reference areas may be the important reason for lesser increase in the prevalence of diabetes and hypertension in interventional areas in comparison with the reference area.

Our study showed more favorable improvement in the Awareness of Hypertension in patients living in interventional than in reference area. As well as better improvement in the treatment rate of hypertension in the overweight and Control rate in the obese hypertensive patients in interventional area compared with reference area.

Many studies showed significant positive correlation between BMI and systolic or diastolic BP in the whole population [23-25]. In our study, the prevalence of hypertension increased by rising BMI in general population in both intervention and reference counties. Obese individuals have approximately 3 times more chance to be hypertensive than those with optimal weight patients furthermore the awareness and treatment rates in obese patients were more than those with optimal weight but the over weight and obese patients could not control their hypertension as well as normal weight participant (table 4). This might be due to poor compliance of obese patients in pharmacological and non-pharmacological treatment.

In the National Health Interview Survey, 38% of women 65 to 74 years old reported hypertension compared with 31% of men in the same age group [17]. Hajjar and colleagues showed that men experienced more hypertension than women (18.1% vs. 10.1%) [14]. In our study the prevalence of hypertension was not significantly different in terms of gender, this finding is contrary to some other surveys in which the prevalence of hypertension has been generally higher among males than females [13]. In our study, awareness, treatment and control of hypertension in women were approximately 2 times greater than men and this indicated that women had more concerns about their self-care. Also our study like other study showed that the prevalence, awareness, treatment, and control rate of hypertension in educated patients is better (table 4). The main goals of interventional activities in this study were life-style modification and increasing the awareness of the community for better detection of hypertension and encouragement of hypertensive patients and their families to improve their knowledge attitudes and behaviors toward treatment and control of their diseases. Education of health professionals toward the importance of pharmacological and non-pharmacological treatments of hypertension was another objective.

## Conclusion

In general the activities of this community trial helped in improving hypertension prevalence, awareness, treatment and control which may lead to decrease in cardiovascular complications in the future. Iran has one of the youngest populations worldwide; therefore we need more efforts to prevent hypertension from early life to improve the control rate of hypertension in young people in order to reduce burden of cardiovascular events in the future. The findings of this large sample size, quality controlled and comprehensive integrated interventions of IHHP can be generalized to other developing countries; however further steps need to be taken to evaluate the effects of these interventional activities on late-onset complications of hypertension.

## Abbreviations

NCDs: Non-communicable diseases; IHHP: Isfahan Healthy Heart Program; BMI: body mass index; CVD: cardiovascular disease; BP: blood pressure; SBP: systolic blood pressure; DBP: diastolic blood Pressure.

## Competing interests

The authors declare that they have no competing interests.

## Authors' contributions

**AK: **designed the study and drafted this manuscript, **GKM: **carried out the study and drafted the tables. **RK: **revising the manuscript procedures and drafting the manuscript, **SS: **helped in designing the study, **MG: **helped in drafting the manuscript and statistical analysis, **AT: **helped in designing and drafting the manuscript, **FN: **data analysis, **NS: **revising the manuscript. All authors read and approved the final manuscript.

## Pre-publication history

The pre-publication history for this paper can be accessed here:

http://www.biomedcentral.com/1471-2261/10/61/prepub

## Supplementary Material

Additional file 1 **Appendix 1**. Specific interventional strategies for high blood pressure prevention and control in the Isfahan Healthy Heart Program. Click here for file

## References

[B1] Kearney PM Whelton M Reynolds K Muntner P Whelton PK He J Global burden of hypertension: analysis of worldwide data Lancet 2005 365 9455 217 23 15652604 10.1016/S0140-6736(05)17741-1

[B2] Lewington S Clarke R Qizilbash N Peto R Collins R Collins R Age-specific relevance of usual blood pressure to vascular mortality: a meta-analysis of individual data for one million adults in 61 prospective studies Lancet 2002 360 9349 1903 13 10.1016/S0140-6736(02)11911-8 12493255

[B3] Ezzati M Lopez AD Rodgers A Vander HS Murray CJ Selected major risk factors and global and regional burden of disease Lancet 2002 360 9343 1347 60 10.1016/S0140-6736(02)11403-6 12423980

[B4] Chockalingam A Campbell NR Fodor JG Worldwide epidemic of hypertension Can J Cardiol 2006 22 7 553 5 16755308 10.1016/s0828-282x(06)70275-6 PMC2560860

[B5] Cheung BM Ong KL Man YB Lam KS Lau CP Prevalence, awareness, treatment, and control of hypertension: United States National Health and Nutrition Examination Survey 2001-2002 J Clin Hypertens (Greenwich) 2006 8 2 93 8 10.1111/j.1524-6175.2006.04895.x 16470077 PMC8109484

[B6] Wolf-Maier K Cooper RS Banegas JR Giampaoli S Hense HW Joffres M Hypertension prevalence and blood pressure levels in 6 European countries, Canada, and the United States JAMA 2003 289 18 2363 9 10.1001/jama.289.18.2363 12746359

[B7] Gu D Reynolds K Wu X Chen J Duan X Muntner P Prevalence, awareness, treatment and control of hypertension in China Hypertension 2002 40 6 920 7 10.1161/01.HYP.0000040263.94619.D5 12468580

[B8] Choi KM Park HS Han JH Lee JS Lee J Ryu OH Prevalence of prehypertension and hypertension in a Korean population: Korean National Health and Nutrition Survey 2001 J Hypertens 2006 24 8 1515 21 10.1097/01.hjh.0000239286.02389.0f 16877953

[B9] Khosravi A Pourmoghaddas M Kelishadi R Sabet B Ansari R Shirani S Trend in hypertension level, prevalance of hypertension and its care in Isfahan MJIRC 2005 8 2 62 8

[B10] Sarraf-Zadegan N Sadri G Malek AH Baghaei M Mohammadi FN Shahrokhi S Isfahan Healthy Heart Programme: a comprehensive integrated community-based programme for cardiovascular disease prevention and control. Design, methods and initial experience Acta Cardiol 2003 58 4 309 20 10.2143/AC.58.4.2005288 12948036

[B11] Sarrafzadegan N Baghaei A Sadri Gh Kelishadi R Malekafzali H Boshtam M Isfahan healthy heart program: Evaluation of comprehensive, community-based interventions for non-communicable disease prevention Prevention and Control 2006 2 73 84 10.1016/j.precon.2006.10.003

[B12] Grossman E Messerli FH Hypertension and diabetes Adv Cardiol 2008 45 82 106 full_text 18230957 10.1159/000115189

[B13] Shirani Sh Kelishadi R Sarrafzadegan N Khosravi A Sadri Gh Amani S Heidari S Ramezani MA Awareness, treatment and control of hypertension, duslipidemia and diabetes mellitus in an Iranian population East Med Health J 2009 15 6 1474 1483 20218138

[B14] Hajjar I Kotchen TA Trends in prevalence, awareness, treatment, and control of hypertension in the United States, 1988-2000 JAMA 2003 290 2 199 206 10.1001/jama.290.2.199 12851274

[B15] Kannel WB Framingham study insights into hypertensive risk of cardiovascular disease Hypertens Res 1995 18 3 181 96 10.1291/hypres.18.181 7584927

[B16] Nissinen A Tuomilehto J Elo J Alasoini A Varvikko P Puska P North Karelia (Finland) hypertension detection project. Five-year follow-up of hypertensive cohort Hypertension 1983 5 4 564 72 6862580 10.1161/01.hyp.5.4.564

[B17] Cheung BM Ong KL Man YB Lam KS Lau CP Prevalence, awareness, treatment, and control of hypertension: United States National Health and Nutrition Examination Survey 2001-2002 J Clin Hypertens (Greenwich) 2006 8 2 93 8 10.1111/j.1524-6175.2006.04895.x 16470077 PMC8109484

[B18] Maldonado J Blood pressure screening, management and control in England: results from the Health Survey for England 1994 Rev Port Cardiol 1999 18 10 959 60 10590661

[B19] Joffres MR Ghadirian P Fodor JG Petrasovits A Chockalingam A Hamet P Awareness, treatment, and control of hypertension in Canada Am J Hypertens 1997 10 10 Pt 1 1097 102 10.1016/S0895-7061(97)00224-0 9370379

[B20] Vargas CM Ingram DD Gillum RF Incidence of hypertension and educational attainment: the NHANES I epidemiologic followup study. First National Health and Nutrition Examination Survey Am J Epidemiol 2000 152 3 272 8 10.1093/aje/152.3.272 10933274

[B21] Hyman DJ Pavlik VN Characteristics of patients with uncontrolled hypertension in the United States N Engl J Med 2001 345 7 479 86 10.1056/NEJMoa010273 11519501

[B22] Barker WH Mullooly JP Linton KL Trends in hypertension prevalence, treatment, and control: in a well-defined older population Hypertension 1998 31 1 Pt 2 552 9 9453361 10.1161/01.hyp.31.1.552

[B23] Tesfaye F Nawi NG Van MH Byass P Berhane Y Bonita R Association between body mass index and blood pressure across three populations in Africa and Asia J Hum Hypertens 2007 21 1 28 37 10.1038/sj.jhh.1002104 17066088

[B24] Njelekela M Negishi H Nara Y Tomohiro M Kuga S Noguchi T Cardiovascular risk factors in Tanzania: a revisit Acta Trop 2001 79 3 231 9 10.1016/S0001-706X(01)00134-6 11412807

[B25] Kadiri S Walker O Salako BL Akinkugbe O Blood pressure, hypertension and correlates in urbanised workers in Ibadan, Nigeria: a revisit J Hum Hypertens 1999 13 1 23 7 10.1038/sj.jhh.1000722 9928748

