# Supplementary material for: The impact of a 6-year comprehensive community trial on the awareness, treatment and control rates of hypertension in Iran: experiences from the Isfahan healthy heart program
Source: BMC Cardiovasc Disord. 2010 Dec 21;10:61. doi: 10.1186/1471-2261-10-61 (PMC3023732; doi:10.1186/1471-2261-10-61)
Supplement: Additional file 1 — Appendix 1. Specific interventional strategies for high blood pressure prevention and control in the Isfahan Healthy Heart Program. [file 1471-2261-10-61-S1.DOC]

Appendix I. Specific interventional strategies for high blood pressure prevention and control in the Isfahan Healthy Heart Program

|  |  | |
| --- | --- | --- |
| Goals | 1. Improving the knowledge, attitude and behaviors of general population, hypertensive patients and health professionals.  2. Increasing the chance of early detection, treatment and control of hypertension. | |
| Strategies | 1. Educating health professionals (physicians, nurses and health staff) with updates in prevention, screening, diagnosis and treatment of hypertension.  2. Educating the public about prevention, early diagnosis, adherence to treatment and control of hypertension.  3. Offering occasional free blood pressure measurement and cardiovascular risk assessment services to the community in interventional areas. | |
| Interventional activities based on target groups | General Population | - Teaching primary and secondary health care principles of (CVD) prevention and healthy lifestyle such as increasing physical activity, smoking cessation, improving nutrition and target screening through educational programs in media, health camps, schools, worksites, healthy centers along with teaching blood pressure measurement. |
|  | Physicians nurses and health care providers and trainee | - Holding seminars and workshops on the subject of prevention, early diagnosis and treatment of hypertension as well as other cardiovascular risk factors. These have been integrated with the continuous medical education (CME). - Establishing educational cores consisting of physicians, nurses and health care (role models) specialists in health centers and hospitals for teaching their colleagues. - Publishing educational books and local guidelines for physicians, nurses and other healthcare providers. |
|  | Patients and relatives | - Educating hypertension complications and the benefits of treatment. - Discussing different pharmacological and non pharmacological treatments of hypertension. - Educating the importance and how to control other cardiovascular risk factors. - Discussing the importance of compliance of patients by educational programs for patients and relatives. - Educating patient’s relatives’ for screening hypertension and raising their sensitization in favor of the patients’ hypertension treatment. |
